# Systematic Review on the Use of 3D-Printed Models for Planning, Training and Simulation in Vascular Surgery

**DOI:** 10.3390/diagnostics14151658

**Published:** 2024-07-31

**Authors:** Alexandra Catasta, Chiara Martini, Arianna Mersanne, Ruben Foresti, Claudio Bianchini Massoni, Antonio Freyrie, Paolo Perini

**Affiliations:** 1 Vascular Surgery, Cardio-Thoracic and Vascular Department, University-Hospital of Parma, Via Gramsci 14, 43126 Parma, Italy; 2 Department of Medicine and Surgery, University of Parma, Via Gramsci 14, 43126 Parma, Italy; 3 Diagnostic Department, University-Hospital of Parma, Via Gramsci 14, 43126 Parma, Italy; 4 Center of Excellence for Toxicological Research (CERT), University of Parma, 43126 Parma, Italy; 5 Italian National Research Council, Institute of Materials for Electronics and Magnetism (CNR-IMEM), 43124 Parma, Italy

**Keywords:** 3D printing, surgical education, surgical simulation, planning, training, endovascular surgery, vascular surgery

## Abstract

The use of 3D-printed models in simulation-based training and planning for vascular surgery is gaining interest. This study aims to provide an overview of the current applications of 3D-printing technologies in vascular surgery. We performed a systematic review by searching four databases: PubMed, Web of Science, Scopus, and Cochrane Library (last search: 1 March 2024). We included studies considering the treatment of vascular stenotic/occlusive or aneurysmal diseases. We included papers that reported the outcome of applications of 3D-printed models, excluding case reports or very limited case series (≤5 printed models or tests/simulations). Finally, 22 studies were included and analyzed. Computed tomography angiography (CTA) was the primary diagnostic method used to obtain the images serving as the basis for generating the 3D-printed models. Processing the CTA data involved the use of medical imaging software; 3DSlicer (Brigham and Women’s Hospital, Harvard University, Boston, MA), ITK-Snap, and Mimics (Materialise NV, Leuven, Belgium) were the most frequently used. Autodesk Meshmixer (San Francisco, CA, USA) and 3-matic (Materialise NV, Leuven, Belgium) were the most frequently employed mesh-editing software during the post-processing phase. PolyJet™, fused deposition modeling (FDM), and stereolithography (SLA) were the most frequently employed 3D-printing technologies. Planning and training with 3D-printed models seem to enhance physicians’ confidence and performance levels by up to 40% and lead to a reduction in the procedure time and contrast volume usage to varying extents.

## 1. Introduction

Vascular surgery, including both open vascular surgery and endovascular surgery, requires a high degree of precision and skill to ensure the best possible outcomes for patients. Recent advancements in vascular surgery have focused on enhancing precision and skill through the development of smaller guidewires for navigating complex vessels with greater accuracy, and through the introduction of thinner sutures to facilitate more delicate surgical techniques. Moreover, surgeons require extensive anatomical knowledge and proficient technical skills. According to the latest guidelines from the European Society of Vascular and Endovascular Surgery, surgical simulation plays a pivotal role in skill enhancement [1]. Traditional training methods like cadaveric dissection or simulators have limitations in terms of skill acquisition and cost [2]. 

Advances in medical technology have revolutionized the way surgeons are trained and how surgical procedures are planned and executed [3]. The integration of 3D-printed models in vascular surgery shows promise for simulation, planning, and training purposes, enhancing the competence and confidence of surgeons and residents [4].

Even though the body of literature surrounding the use of 3D-printed models in vascular surgery is constantly growing, a comprehensive understanding of the current state of this innovative approach is difficult to achieve. In fact, the literature on this topic is lacking standardization in the imaging, processing software, and application methods. Furthermore, the results concerning applications in training and clinical practice are extremely heterogeneous.

This systematic review explores the diverse applications of 3D-printed models in vascular surgery, with a focus on their potential to transform surgical preparation for complex procedures and simulation of navigation through complex anatomies. The study aims to consolidate the existing literature, analyze the manufacturing process employed for 3D-printed models and evaluate the simulation methodologies, assessing the impact of 3D models on vascular surgery education and practice. Ultimately, the goal is to offer comprehensive insights into the use of 3D printing in vascular surgery, aiding surgeons in selecting appropriate materials and printing methods based on their intended applications.

## 2. Materials and Methods

### 2.1. Data Sources, Search Strategy and Selection Criteria

This study was undertaken in accordance with the Preferred Reporting Items for Systematic Reviews and Meta-Analyses (PRISMA) statement [5]. Papers about 3D-printed models for simulation, planning and training in vascular surgery were searched for in PubMed, Web of Science, Scopus and in the Cochrane Library. We performed the last search on 1 March 2024, with no restrictions on the initial date of the studies included. The following words were searched in PubMed: (“planning” OR “training” OR “simulation”) AND (“endovascular” OR “vascular”) AND (“surgery” OR “treatment” OR “therapy”) AND (“3D printing” OR “3D print” OR “bioprint” OR “fabrication” OR “biofabrication” OR “printing”). The database research was undertaken by 3 authors (AC, AM, and CM), independently. Controversies were solved by the senior author (PP). Titles and abstracts were screened to exclude irrelevant or duplicate abstracts. We included papers that reported the outcome of applications of the 3D-printed models, excluding case reports or very limited case series (≤5 printed models or tests/simulations) in order to reduce the methodological or small sample bias and to report and analyze papers about validated techniques. Non-English articles were included when an English abstract with extractable data was provided. The inclusion criteria were (i) simulation, training or planning regarding the treatment of vascular diseases (stenotic/occlusive or aneurysmatic), (ii) use of 3D-printing technology, and (iii) reported technical and/or training success. The additional exclusion criterion was different areas than vascular surgery (cardiac or intra-cranial vessels). In case of papers originating from the same database, we considered only the most recent version for data extraction.

### 2.2. Data Extraction, Outcome Measures, and Evaluation of Study Quality

Three authors independently conducted the data extraction and evaluated the quality of the studies (AC, AM, and CM). Controversies were resolved by a fourth author (PP), who did not participate in the aforementioned process. The extracted data included the first author, year of publication, study type (retrospective, prospective, or feasibility study), surgical planning and training methods, surgical performance and self-confidence of residents/surgeons, vascular disease classification, details of 3D printing (materials, imaging software, types of printing, printed models, printing time, and model costs), pre-printing imaging modalities (computed tomography/magnetic resonance with or without contrast medium), anatomical area of surgical training, complications, and effectiveness of the planning (technical success). Data that could not be inferred were labeled as “not extractable” (NE) or “not reported” (NR), as appropriate. The quality of the studies was assessed using the Mixed Methods Appraisal Tool (MMAT) [6]. The records were assessed independently by two reviewers (PP, CBM) using a rating of “good” (≥6 “Yes” answers), “moderate” (4–5 “Yes” answers) or “low” (1–3 “Yes” answers) quality agreed between them. We focused on the applications of 3D-printed models and their outcomes in vascular surgery. Therefore, the main aspects and outcomes considered were as follows:

- Diagnostic imaging technique;
- Image processing and post-processing software;
- 3D-printing technologies and materials;
- Feasibility of 3D-printing technology application in vascular surgery;
- 3D-printed models in vascular training;
- 3D-printed models in vascular planning.

### 2.3. Definitions

The planning was defined as the selection of the most suitable material and the exploration of various possible treatments and/or strategies for different vascular diseases and patients. This planning process should rely on the analysis of 3D-printed custom-made models. Training, on the other hand, was described as the simulation of interventions by residents or surgeons using these 3D models (with or without subsequent, direct application on the patient). This simulation is intended to enhance their technical skills. Both planning and training were highlighted as tools to enhance the competence and confidence of vascular surgeons. We interpreted performance as the enhancement of technical skills and self-confidence as the optimal security during operations. 

## 3. Results

### 3.1. Review Design and Baseline Characteristics

**Figure 1:**
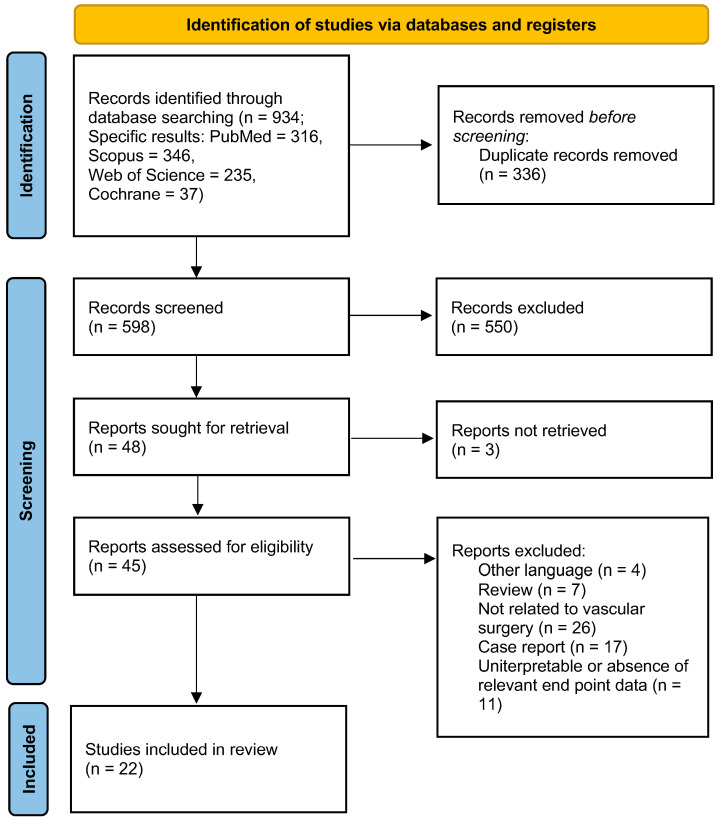
PRISMA flow diagram showing the process used to identify the included studies. PRISMA, Preferred Reporting Items for Systematic Reviews and Meta-Analyses.

The initial research identified 934 papers. After removing 336 duplicates, 598 papers were screened. Of these, 550 were excluded based on their title and abstract. This left 48 papers, of which 45 were retrievable and were therefore reviewed in full-text form. After the exclusion of non-English papers (*n* = 4), reviews (*n* = 7), articles not related to vascular surgery (*n* = 26), case reports (*n* = 17), and papers with uninterpretable or unextractable data (*n* = 11), 22 studies about the use of 3D-printed models for simulation-based training and/or planning in vascular surgery were finally included in the systematic review (Figure 1). A total of 15 analyzed papers were feasibility studies [7,8,9,10,11,12,13,14,15,16,17,18,19,20,21], 5 were prospective studies [22,23,24,25,26], and 2 were retrospective [27,28]. All the papers were focused on vascular diseases (Table 1): 15/22 on aneurysm [7,9,10,11,13,14,15,17,18,20,22,23,24,25,26], 4/22 on steno-occlusive pathology [12,19,21,22,28], 1 on both aneurysm and stenotic/occlusive pathology [8] and in 2 papers this difference was not reported [16,27]. All the studies concerned vascular diseases (carotid, thoraco-abdominal, or visceral arteries), predominantly focused on the thoraco-abdominal pathology of the aorta (17/22) [7,8,10,11,13,14,15,16,17,18,20,22,23,24,26,27]. According to the MMAT [6] and the agreed rating, 17 [7,8,9,10,11,12,15,16,18,19,21,22,23,25,26,27,28] studies were considered to be of good quality, 1 of moderate quality [13], and 4 studies of poor quality [14,17,20,24] (Supplementary Table S1). 

### 3.2. Diagnostic Imaging

A total of 12 out of 22 studies reported using and exporting computed tomography (CT) angiography data in DICOM (Digital Imaging and Communications in Medicine) format [7,10,11,14,19,21,22,23,24,25,26,28]. The remaining 10 studies did not specify the data export format [8,9,12,13,15,16,17,18,20,27]. Reconstruction with a thickness of 1 mm was the most frequently reported [7,9,10,20,21,22,23,26,27,29]. Three studies reported a thickness ranging from 0.5 to 0.6 mm [17,18,24]. Nine studies did not specify the thickness of the reconstructed layer [8,11,12,13,14,15,16,25,28]. Two studies did not utilize CT data to generate patient-specific 3D models, opting instead for the creation of generic 3D models for simulation [12,21]. Only one study used both contrast-enhanced magnetic resonance imaging and CT [10]. 

### 3.3. Software for 3D Model Generation

**Figure 2:**
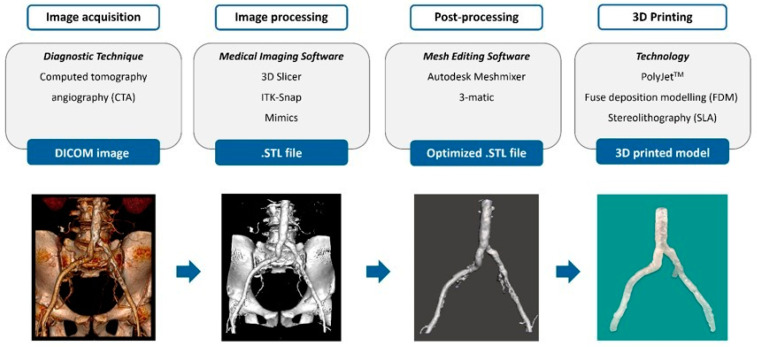
Entire process from CTA image acquisition to 3D model printing, reporting the most used technologies in our review for each step.

The process of generating 3D patient-specific models starts with the acquisition of patients’ CT images in the form of DICOM datasets (Figure 2). 

A medical-imaging software (MIS) is then utilized to isolate the vascular region of interest (ROI) via image segmentation and execute a 3D reconstruction of the segmented region. Subsequently, the visualized 3D model is usually exported as a .STL file [7,8,10,11,12,13,14,15,16,17,18,19,20,21,22,24,25,27,28]. This file could be directly sent for printing. However, to correct errors resulting from the semi-automated segmentation techniques, mesh-editing software (MES) is employed to produce an optimized .STL file through mesh refining, surface smoothing, and model scaling.

The included studies utilized various open-source or commercially available software (Table 2). For MIS, among the open-source options, 3D Slicer [30] in 2/22 studies [18,20] and in 2/22 [13,22] ITK-Snap [31] emerged as the most employed tools [10,11,14,23]. Among the commercial software, Mimics (Materialise NV, Leuven, Belgium) in 4/22 [10,11,23] studies and OsiriX in 2/22 studies [9,15] were the predominant choices. For MES, the most used among the open-source options was Autodesk Meshmixer (San Francisco, CA, USA) in 8/22 [12,16,17,18,19,20,24,32], while 3-matic (Materialise NV, Leuven, Belgium) was the main choice among the commercial software in 2/22 [11,14]. Four studies utilized advanced image analysis software such as Syngo.Via (Siemens Healthineers, Erlangen, Germany) [7], 3-matic [10] and MATLAB (MathWorks, Natick, Massachusetts, USA) [9,13] to perform accuracy measurements on the CT-scanned 3D-printed models. Two studies used computer-aided design (CAD) software to create the 3D models (not starting from a DICOM dataset) [12,21].

### 3.4. 3D-Printing Technology in Vascular Surgery

Eighteen articles mentioned the model and the brand of 3D printer used [7,8,9,10,11,12,13,14,15,16,18,19,20,21,22,24,25,27,28]. Stratasys (Eden Prairie, MN, USA) and 3D Systems Corporation (Rock Hill, SC, USA) were the most frequently mentioned suppliers (Table 3).

**Figure 3:**
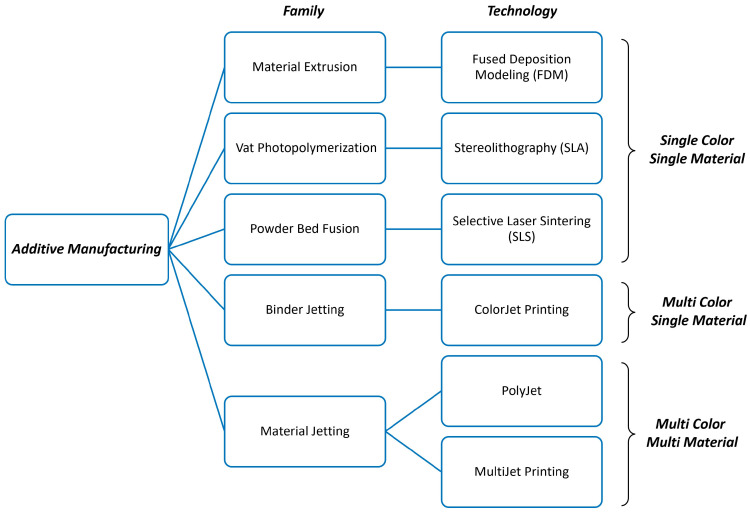
3D-printing technologies and families. To note, 3D printers originally engineered for single-color, single-material applications (such as FDM, SLA, SLS, and ColorJet technologies) can be adapted into machines capable of multi-color, multi-material printing by integrating supplementary accessories.

The time taken to print the model ranged from 3 to 72 h, depending on the printed region volume and the technology [7,8,9,10,11,12,13,16,17,18,24]. The cost of a single model ranged from EUR 10 to EUR 1500, depending on the materials and the printing technology [11,12,14,17,20,21,22,24,25,28]. The most frequent types of 3D-printing technology used were Fuse Deposition Modelling (FDM) [9,10,15,18,19,20,24,25], Stereolithography (SLA) [10,12,20,21,23,25,27,28], and PolyJet [7,10,11,13,14,16,20,25]. The other technologies were MultiJet [20], ColorJet [8,15,22] and Selective Laser Sintering (SLS) (Figure 3) [10].

The materials used to print the 3D model varied on the basis of the printing technology. PolyJet and SLA use different photopolymer resins, respectively, polyurethane (PUR)-based [7,10,11,13,14,16,20,25] and methacrylate-based resins [10,12,20,21,23,25,27,28]. FDM uses thermoplastic materials such as PLA (polylactic acid) [8,10,15,22,25] and ABS (acrylonitrile butadiene styrene) [17,20], often covered with silicone [8,17,19,22,25]. The hardness of a 3D model can range from flexible to rigid. In multi-material printers, the model can incorporate both flexible and rigid materials. The model surface can be opaque or translucent (depending on the surface roughness), and the model appearance can be transparent or not. A total of 13 studies generated rigid and opaque models (13/22) [7,8,10,11,13,14,15,17,18,20,22,25,29]; in 8 studies, the models were rigid and transparent (8/22) [12,20,21,23,24,25,26,28]; in 5, the models were flexible and transparent (4/22) [20,24,25,27]; in 5, they were flexible and opaque (5/22) [10,16,20,24,25]; and in 4, there was a combination of flexible and rigid materials with opaque appearance (4/22) [7,11,13,32]. Overall, 21 articles out of 22 reported the high accuracy of the generated 3D models. These data are reported in Table 4. The dimensional accuracy was established by means of different methods. Shibata et al. compared the whole shapes of the segmented aneurysm and related arteries from patient CTA and model data using the Dice coefficient index (a measure of the model anatomic accuracy) [9]. In other cases, the models were deemed to be accurate, as their production had been overseen by endovascular operators, some models had been rescanned, and their accuracy was determined by fusing the CT of the model to the host dataset [15]. O’Hara et al. [16] used X-ray and digital subtraction angiography to verify the accuracy and compatibility of the small vessels in the 3D-printed model. The slice thickness and the space between the slices are determinant aspects, where larger gaps between slices or thicker slices reduce the accuracy of the produced models [10,13].

### 3.5. Feasibility of 3D-Printing Technology for Vascular Models

Six studies focused on demonstrating the feasibility of employing 3D-printing technology to create patient-specific 3D models for vascular surgery applications in a hospital setting [7,9,10,14,16,20]. Among these, four created abdominal aortic aneurysm (AAA) 3D models [7,10,14,20]. One study generated 3D models of visceral artery aneurysms [9] and one study explored various vascular anatomical regions, including the carotid artery [16]. All six studies detailed the process used to generate the 3D-printed vascular models. Images of the CT-scanned 3D-printed models were used to statistically calculate the difference in the size and shape of the models compared to patient CTA images using different software (reported in Table 2). One study utilized MATLAB (MathWorks, Natick, MA, USA), a mathematical computing software [9], whereas another employed Syngo.Via (Siemens Healthineers, Erlangen, Germany), a medical-imaging software known for its advanced image analysis capabilities [7]. One study utilized 3-matic (Materialise NV, Leuven, Belgium), a mesh-editing software, and also performed manual analysis using Vernier calipers [10]. One study optimized the process methodology to prove the feasibility of creating complex geometries and small diameter vessels [16].

### 3.6. 3D-Printed Models in Vascular Surgery Training

Eleven studies utilized additive manufacturing to generate 3D-printed models for simulation-based training in vascular surgery (Table 5). Five developed 3D AAA models and simulated the endovascular aneurysm repair (EVAR) technique in different ways [10,11,17,18,25]. One of these studies utilized 3D AAA models to facilitate the explanation of the pathology to patients [17]. Four studies generated 3D-printed vascular phantoms to mimic peripheral arterial disease (PAD) and practice endovascular interventions [8,19,21,28]. One study simulated ultrasound-guided femoral artery access [8], whereas three studies simulated the percutaneous transluminal angioplasty technique [19,21,28]. Two studies generated 3D-printed vascular models for practicing embolization techniques [12,27]. 

Nine studies employed pre-operative CTA images to generate patient-specific 3D-printed vascular models [8,10,11,17,18,19,25,27,28]. Two studies opted for a different approach, designing and developing a 3D-printed simulator to mimic specific pathologies (specifically, a simulator for endovascular embolizations, and a simulator for peripheral artery disease endovascular treatment) [12,21]. An endovascular simulation setup was designed and fabricated in seven studies by integrating the 3D vascular model with a fluid pump to establish a closed, pulsatile circulatory system [8,10,11,17,19,27]. Five studies out of eleven performed the simulation under fluoroscopy [10,11,17,18,28]. 

Five studies employed different methods to evaluate the 3D-printed model or the designed simulator. Four studies utilized questionnaires to gather operators’ opinions, assessing both the realism of the developed system and the effectiveness of the training sessions [8,12,21,25]. One involved the use of a rating scale to evaluate the realism and the operator perception of the setup [27]. Three studies reported specific data on the dimensional accuracy of the 3D-printed models, comparing the 3D anatomical vascular measurements with real-life data [10,25,28]. Göçer et al. and Nguyen et al. confirmed the measurements with a caliper [10,28]. Torres et al. compared the measurement of the total length from the lowest renal artery to the iliac bifurcation on the 3D model with intraoperative measures (obtained with vessel-sizing catheters) [25]. Among the 11 studies reviewed, 5 evaluated the effects of simulation-based training on residents [12,17,18,21,25]. Specifically, three studies demonstrated improvements in residents’ surgical performance and self-confidence following training [12,17,21,25]. Self-confidence was assessed through the completion of questionnaires or surveys [12,21,25]. Performance was evaluated based on objective parameters, such as the procedure duration, calculated during simulation [12,21] or during real interventions, comparing residents trained with and without the 3D model [25]. One study documented the experience of a trainee performing anastomosis on a 3D-printed AAA model [17], while another involved deploying a bifurcated aortic endograft in a 3D-printed AAA model under fluoroscopy [18], guided by a consultant in both instances. One study only demonstrated that there is a correlation between the operator’s training outcome and previous experience by comparing the performance of less- and more-experienced operators [11]. 

### 3.7. 3D-Printed Models in Vascular Surgery Planning

Planning using 3D-printed models has been successfully employed in various vascular procedures, as documented in 8 out of 22 studies [13,15,17,22,23,24,25,26]. In one study, 28 surgeons retrospectively reviewed 6 cases of complex aortic aneurysm, assessing that the 3D model guided changes in 20% of the surgical planning, switching from EVAR to open repair, or from off-the-shelf solutions to custom-made aortic endografts, with self-confidence increasing in 40% of cases [15]. Seven studies prospectively used 3D models for pre-operative planning, including a total of 203 patients [13,17,22,23,24,25,26]. One demonstrated that residents training with patient-specific 3D models before EVAR increased their peri-operative self-confidence and improved the objective surgery metrics, such as reduced fluoroscopy and procedure times, lower contrast volumes used, and quicker target vessel cannulation [25]. Two studies reported an increase in self-confidence [17,22], with one also reporting a significative reduction in the operating time [22], in both cases without detailing the assessment methods. In two studies, the 3D-printed models were used to accurate position the fenestrations in fenestrated-EVAR (FEVAR) [23,26]. One study compared the 3D in vitro test with numerical simulation (NS), finding equivalent accuracy but a shorter FEVAR device delivery time in favor of NS [26]. The other reported that 20% of fenestrated custom-made stent-grafts were modified after testing prototypes in a 3D-printed aortic model [23]. In one study using both virtual and 3D-printed models, it was reported that 3D models helped redefine the surgical approach and simulate device deployments. The study suggested the complementary use of these tools to enhance the depth perception and dynamic representation [24]. One article reported the experience of using 3D models in abdominal vascular surgery with a robotic approach [13]. Additionally, two out of seven articles stated that the procedures achieved successful technical outcomes and were completed without complications [22,23].

## 4. Discussion

The rapid evolution of image-processing software and 3D printers has made patient-specific 3D models accessible and cost-effective tools, sparking increased interest in 3D printing for vascular surgery [21,33,34]. These models have been evaluated for their potential to improve pre-operative planning and simulation in both open and endovascular procedures, aiding in complex vascular disease diagnosis and treatment pathways [23,24,25]. Additionally, given the necessity of incorporating simulation-based training in residency programs and the challenge of the high costs associated with traditional simulators, there is growing exploration of 3D-printed models as tools to develop residents’ surgical technical skills [1]. Traditional training methods such as cadavers are non-reproducible due to the anatomical variability and limited availability; while they are useful for hands-on training, cadavers are less effective for procedural planning as they lack the ability to simulate specific patient anatomies [2]. However, challenges remain in establishing standardized methodologies for both training and planning purposes [21,35,36].

The 22 selected studies provided a comprehensive overview of 3D-printing applications in vascular surgery, encompassing the technology, materials used, production times, costs, and the quality of imaging data utilized. They showed that the use of 3D-printed models is more frequent in aneurysmal pathology compared to steno-occlusive pathology. This is likely due to the current need for more planning in complex EVAR cases. Moreover, large vessels, and in particular AAA, are probably easier to print since there is no need to reproduce very narrow lumens or even occlusions as for PAD patients. In this context, patient-specific 3D-printed vascular models are used in simulations either independently or connected to a fluid pump to accurately replicate pulsatile circulation [8,10,11,17,27,29]. Common 3D-printing technologies like FDM and SLA are cost-effective and primarily designed for single-material, single-color prints, offering a good balance between cost and precision. Advanced techniques like PolyJet technology can simultaneously use multiple materials and colors, resulting in superior print quality with a high geometric accuracy, but at a higher cost. The higher costs are due not only to different technologies and complexity but also to equipment and maintenance, which increases the overall cost of the printing services. 

Additionally, the cost of 3D-printed models is closely related to the choice of materials, which depends on the anatomical features being reproduced. PolyJet technology uses polyurethane-based resins, offering a wide range of material choices and allowing for the replication of complex anatomical features. However, these resins are more expensive compared to the polylactic acid (PLA) or acrylonitrile butadiene styrene (ABS) used in FDM, or the methacrylate-based resins used in SLA. PLA and ABS are cost-effective options for high-resolution prints with choices in terms of the flexibility, hardness, and transparency. However, they have fewer material options and generally cannot simultaneously use multiple colors or materials in a single print. Therefore, it is essential to carefully evaluate the specific needs of the project or final application to achieve an optimal balance between quality and costs.

Rigid materials for 3D models are useful for understanding the anatomy of the vessels to be treated or for performing accuracy measurements. To plan and simulate an endovascular procedure, it may be more appropriate to adopt elastic, flexible, and transparent models to allow the direct visualization of the endovascular materials, such as guidewires or catheters [7,10,20,23,24,25,27,28]. Simulation of stent-graft deployment in a nonrigid model may also provide an assessment of the behavior and deformation of the aortic wall [26]. The production time depends on the type of 3D model to be printed (dimensions and materials), as well as on the type of 3D-printing technology. However, in most of the analyzed articles, it was not clear if the reported time for creating the 3D model related to only the printing time or to all of the production process. 

The accuracy of 3D-printed models has proven to be critical for surgeons making clinical decisions and studies have evaluated it using various methods. Alongside the printing technology, accuracy is highly dependent on the quality and resolution of the original CT imaging, preferably with submillimetric slice thicknesses. The choice of software used in the manufacturing process also plays a significant role. Open-source software offers accessible tools for CT image processing, .STL file generation and post-processing, while commercial software suites like those produced by Materialise provide advanced and semi-automatic features specifically designed for medical use. These include sophisticated algorithms for segmentation and model optimization, potentially leading to higher-quality outcomes. 

Moreover, 3D-printing technology is mostly used to train residents in endovascular procedures like EVAR, lower-extremity arterial interventions, and embolization techniques. Trainees can familiarize themselves with the numerous materials and different techniques in vascular and endovascular surgery. 

It was reported that pre-operative planning was changed after the evaluation of 3D-printed models, particularly when complex endovascular techniques such as the chimney-EVAR were required [22]. The 3D-printed models were also reported to be a useful aid for modifying custom-made grafts: in some cases, the design model was modified from its original prototype based on the surgeon’s instructions based upon the measurements performed on the 3D-printed model [23]. The 3D models can also be helpful in reducing the dose of contrast medium, as well as the fluoroscopy and operating time [22,25]. In fact, the surgeon will likely have already acquired the tactile sensations regarding stenosis or vessel tortuosity of the specific patient. In some articles, the model is tested in training under X-rays [11,17,18,20,24,28]. To avoid the risks of radiation exposure, the use of transparent models is advisable, which are used by most of the authors. Transparent models can be used in association with a light-emitting diode (LED) light and a camera connected with a screen to mimic the operatory room environment and the two-dimensional fluoroscopy images [12,25,27]. By adopting this method, the 3D model is projected onto the screen in two dimensions, and the result is very similar to what is obtained in the angiography suite. The pre-operative use of 3D-printed models was also deemed crucial in robotic surgery, where tactile feedback is absent [13].

Furthermore, 3D-printed patient-specific anatomical models can reproduce in detail all the anatomical structures that can identify possible variations or anomalies and sometimes aid in anticipating surgical risks. These models, by providing tactile feedback, enhancing 3D visualization, and improving understanding of anatomy, have been shown to improve residents’ skills and increase their self-confidence [12,17,18,22,25]. Furthermore, planning and practicing surgical procedures using patient-specific 3D-printed models has been shown to improve operative performance. This has the potential to reduce peri-operative complications, thereby enhancing patient safety [22,25].

The main limitation of this systematic review is that the included papers presented highly heterogeneous data about the materials, methods and purposes, and they were therefore challenging to compare. Data regarding the operating times are often lacking or incomplete [22]. Thus, even though it was reported as an advantage of the use of 3D-printed models, it was not possible to perform a quantitative synthesis of the operating time reduction. Despite the majority of the articles reporting a reduction in operative complications, the type of complication is rarely described [17,23,26,32]. And, above all, it is not reported if any complication was possibly related to the 3D-printed model. Finally, there is currently no standard for evaluating patient-specific 3D-printed vascular models, even though all the included articles reported positive evaluation of the 3D model.

The refinement and personalization of 3D-printed models hold immense potential in the foreseeable future. Customization of 3D-printed anatomical replicas based on patient-specific data already offers the promise of tailoring training and surgical planning to each individual case, enhancing the precision in surgical procedures. Moreover, advancements in materials and printing techniques may lead to more anatomically accurate and physiologically responsive models, enabling a closer simulation of the dynamic conditions encountered during vascular surgeries [37]. Further research in this area promises to not only advance surgical education but also to improve patient outcomes and safety in the field of vascular surgery.

## 5. Conclusions

Simulation-based training and planning using patient-specific 3D-printed models are gaining ground in vascular surgery, showing promising future prospects. These models, whether with or without a designed simulation setup, can be produced affordably and with high dimensional accuracy, allowing surgeons to accurately replicate real-life procedural challenges. In the foreseeable future, they have the potential to be recognized as essential tools for aiding in pre-operative planning and advancing residents’ surgical technical skills. However, establishing standardized methodologies for generating and validating 3D-printed models in terms of the accuracy and effectiveness is necessary for their integration as a clinical standard.

## Figures and Tables

**Table 1:** Anatomical regions considered for 3D printing in the included studies.

Author	Year	Carotid Arteries	Thoraco-Abdominal	Infrainguinal Arteries	Visceral Arteries	Aneurysm Disease	Steno-Occlusive Disease
Foresti [21]	2024			X			X
Nguyen [10]	2023		X			X	
Kaufmann [27]	2022		X		X		
Magagna [17]	2022		X		X	X	
Little [18]	2022		X			X	
Göçer [28]	2021			X			X
Matyjas [12]	2021						X
Kliewer [26]	2021		X		X	X	
Kaschwich [7]	2021		X			X	
Coles-Black [20]	2021		X			X	
Kaschwich [19]	2020			X			X
Borracci [24]	2020		X	X	X	X	
Kärkkäinen [11]	2019		X			X	
Marconi S. [13]	2019		X			X	
Bortman [14]	2019		X			X	
Marone [22]	2018		X		X	X	
Shibata [9]	2017				X	X	
Taher [23]	2017		X		X	X	
Torres [25]	2017		X			X	
Tam [15]	2016		X			X	
O’Hara [16]	2016	X	X				
O’Reilly [8]	2015		X		X	X	X

**Table 2:** Software characteristics and utilization in 3D models’ manufacturing process.

Software	Supplier	Category	Model Design	Image Processing/3D Reconstruction	.STL File Generation	.STL File Post-Processing	Author
Autodesk fusion 360	Autodesk, Inc. (San Francisco, CA, USA)	CAD/CAM	x		x	x	Matyjas [12]
SolidWorks^®^ v. 2015	Solidsolution (Vélizy-Villacoublay, France)	CAD	x		x		Foresti [21]
Mimics	Materialise NV (Leuven, Belgium)	MI		x	x		Nguyen [10]; Kärkkäinen [11]; Bortman [14]; Taher [23]
OsiriX	Pixmeo (Geneva, Switzerland)	MI		x	x		Shibata [9]; Tam [15]
3D Slicer	Open-source (www.slicer.org)	MI		x	x		Little [18]; Coles-Black [20]
ITK-Snap	Open-source (http://www.itksnap.org/)	MI		x			Marconi [13]; Marone [22]
ImageJ	Open-source (https://imagej.nih.gov/ij/index.html accessed on 1 March 204)	MI		x	x		Kaufmann [27]
InVesalius	Open-source (https://www.cti.gov.br/invesalius/ accessed on 1 March 2024)	MI		x	x		Magagna [17]
Mimics Innovation Suite	Materialise NV (Leuven, Belgium)	MI		x	x		Göçer [28]
Vascular Modelling Toolkit	Open-source (http://www.vmtk.org/)	MI		x	x	x	Marconi [13]
TeraRecon iNtuition Unlimited	TeraRecon (Durham, NC, USA)	MI		x	x		Torres [32]
Vitrea 3D Station	Vital Images, Inc. (Minnetonka, MN, USA)	MI		x	x		O’Hara [16]
Syngo.via *	Siemens Healthineers (Herlangen, Germany)	MI		x	x		Kaschwich [19,29]
Blender	Open-source (www.blender.org)	ME	x		x	x	Kaufmann [27]
Meshmixer	Open-source (San Francisco, CA, USA)	ME	x			x	Little [18]; Magagna [17]; Matyas [12]; Coles-Black [20]; Kaschwich [19,29]; Borracci [24]; Torres [25]; O’Hara [16]
3-matic	Materialise NV (Leuven, Belgium)	ME				x	Nguyen [10]; Kärkkäinen [11]; Bortman [14]
Meshlab	Open-source (www.meshlab.net)	ME				x	Marconi [13]
Magics	Materialise NV (Leuven, Belgium)	AM				x	Torres [25]
Netfabb	Autodesk, Inc. (San Francisco, CA, USA)	AM				x	Marone [22]
Slic3r	Open-source (https://slic3r.org/)	AM			x	x	Foresti [21]
MATLAB *	MathWorks, Inc. (Natick, MA, USA)	MC					Marconi [13], Shibata [9]

Abbreviation: 3D = three-dimensional; STL = stereolithography; CAD = computer-aided design; CAM = computer-aided manufacturing; MI = medical imaging; ME = mesh editing; AM = additive manufacturing; MC = mathematical computing. * Software used for advanced accuracy measurements on CT images of 3D-printed models.

**Table 3:** The suppliers of the different printers.

Printer	Suppliers
CubePro	3D Systems Corporation (Rock Hill, SC, USA)
ProJet 3500	3D Systems Corporation (Rock Hill, SC, USA)
Projet460 Plus	3D Systems Corporation (Rock Hill, SC, USA)
ZPrinterVR 250	3D Systems Corporation (Rock Hill, SC, USA)
sPro 60	3D Systems Corporation (Rock Hill, SC, USA)
Felix 3	FELIXprinters (Ijsselstein, The Netherlands)
Form 1+	Formlabs (Somerville, MA, USA)
Form 2	Formlabs (Somerville, MA, USA)
Form 3	Formlabs (Somerville, MA, USA)
Ultimaker S3	Ultimaker B.V. (Utrecht, The Netherlands)
Ultimaker S5	Ultimaker B.V. (Utrecht, The Netherlands)
MakerBot Replicator 2X	Stratasys (Eden Prairie, MN, USA)/MakerBot (New York City, NY, USA)
Objet260 Connex3	Stratasys (Eden Prairie, MN, USA)
Objet30 Prime	Stratasys (Eden Prairie, MN, USA)
Stratasys J750	Stratasys (Eden Prairie, MN, USA)
J750 Digital Anatomy	Stratasys (Eden Prairie, MN, USA)
Objet350 Connex	Stratasys (Eden Prairie, MN, USA)
Objet500 Connex3	Stratasys (Eden Prairie, MN, USA)
Objet Eden 260V	Stratasys (Eden Prairie, MN, USA)
FlashForge Creator Pro	Flashforge (Zhejiang, China)
Prusa i3 MK3S+	Prusa Research (Prague, Czech Republic)
ZPrinter 450	Z Corporation (3D Systems Corporation, Rock Hill, SC, USA)
Orcabot 3D printer	Mendel-Parts (Prodim International, Helmond, The Netherlands)

**Table 4:** Specificities and characteristics of the 3D-printed model in each included study.

Author	Year	Printer	3D-Printing Technology	Model Material	Model Hardness	Model Appearance	Printing Time (h)	Cost (EUR)	Accuracy *
Foresti [21]	2024	Form 2	SLA	M-based resin	rigid	transparent	21	200	high
Nguyen [10]	2023	Ultimaker S5	FDM	PLA	rigid	opaque	n.a.	n.a.	high
		sPro 60	SLS	nylon	rigid	opaque	n.a.	n.a.	high
		J750 Digital Anatomy	PolyJet	PUR-based resin	(1) rigid; (2) flexible	opaque	n.a.	n.a.	high
		Form 3	SLA	M-based resin	rigid	opaque	n.a.	n.a.	high
Kaufmann [27]	2022	Form 3	SLA	M-based resin	flexible	transparent	n.a.	low	high
Magagna [17]	2022	n.a.	n.a.	silicone	rigid	opaque	24–72	1000–1500	high
Little [18]	2022	Ultimaker S3	FDM	PVA	rigid	opaque	n.a.	100	high
Göçer [28]	2021	Form 2	SLA	M-based resin	rigid	transparent	6	400	high
Matyjas [12]	2021	Form 2	SLA	M-based resin	rigid	transparent	8	low	high
Kliewer [26]	2021	External provider **	n.a.	n.a.	rigid	transparent	n.a.	n.a.	high
Kaschwich [7]	2021	Objet500 Connex3	PolyJet	PUR-based resin	flexible + rigid	opaque	n.a.	n.a.	high
Coles-Black [20]	2021	Objet500 Connex3Stratasys J750ProJet 3500	PolyJet	PUR-based resin	flexible	transparent	n.a.	650–930	high
		Form 2	SLA	M-based resin	rigid or flexible	opaque or transparent	n.a.	50–100	high
		FlashForge Creator Pro Prusa i3 MK3S + Ultimaker S5MakerBot Replicator 2X	FDM	ABS	rigid	opaque	24–48	10–20	high
Kaschwich [29]	2020	Felix3	FDM	silicone	rigid	opaque	n.a.	low	n.a.
Borracci [24]	2020	External provider ^§^	FDM	n.a.	rigid or flexible	opaque or transparent	n.a.	90–460	high
Kärkkäinen [11]	2019	Objet500 Connex3	PolyJet	PUR-based resin	flexible + rigid	opaque	24–36	280–370	high
Marconi [13]	2019	Objet260 Connex3	PolyJet	PUR-based resin	flexible + rigid	opaque	10	n.a.	high
Bortman [14]	2019	Objet30 Prime	PolyJet	PUR-based resin	rigid	opaque	3	30	high
Marone [22]	2018	Projet460 Plus	ColorJet	silicone	rigid	opaque	8	100–150	high
Shibata [9]	2017	CubePro	FDM	nylon	rigid	n.a.	n.a.	low	high
Taher [23]	2017	External provider *	SLA	M-based resin	rigid	transparent	n.a.	n.a.	high
Torres [25]	2017	Form 1+	SLA	M-based resin	flexible	transparent	n.a.	150	high
		MakerBot Replicator 2X	FDM	silicone	rigid	opaque	n.a.	120	high
		Objet350 Connex	PolyJet	PUR-based resin	(1) flexible; (2) rigid; (3) flexible + rigid	(1) opaque; (2) transparent; (3) opaque	n.a.	475	high
Tam [15]	2016	ZPrinter 450	ColorJet	plaster	rigid	opaque	24	185	good
		Orcabot 3D printer	FDM	PLA	rigid	opaque	24	185	high
O’Hara [16]	2016	Objet Eden 260V	PolyJet	PUR-based resin	flexible	opaque	24	n.a.	high
O’Reilly [8]	2015	ZPrinterVR 250	ColorJet	silicone	rigid	opaque	n.a.	low	high

* Accuracy was self-assessed by each author using different methods. ** Terumo Aortic; ^§^ MIRAI 3D. SLA = stereolithography; FDM = fuse deposition modeling; SLS = selective laser sintering; M = methacrylate; PUR = polyurethane; PLA = thermoplastic polylactic acid; ABS = thermoplastic acrylonitrile butadiene styrene, PVA = polyvinyl alcohol; n.a. = not available.

**Table 5:** Differences in 3D printed models’ utilization in vascular surgery training.

Author	Year	Patient-Specific 3D Model	In-House Designed Set-Up	3D-Printed Model Only	Simulated Technique
Foresti [21]	2023	no	yes	no	PTA
Nguyen [10]	2023	yes	yes	no	EVAR
Kaufmann [27]	2022	yes	yes	no	Endovascular embolization
Magagna [17]	2022	yes	yes	no	EVAR
Little [18]	2022	yes	no	yes	EVAR
Göçer [28]	2021	yes	no	yes	PTA
Matyjas [12]	2021	no	yes	no	Endovascular embolization
Kaschwich [19]	2020	yes	yes	no	DUS guided peripheral endovascular intervention
Kärkkäinen [11]	2019	yes	yes	no	EVAR
Torres [25]	2017	yes	no	yes	EVAR
O’Reilly [8]	2015	yes	yes	no	Femoral artery access with DUS imaging

Abbreviation: PTA: percutaneous transluminal angioplasty, EVAR: endovascular aneurysm repair; DUS: Doppler ultrasound.

## Data Availability

The original contributions presented in the study are included in the article and Supplementary Materials, further inquiries can be directed to the corresponding author.

## Supplementary Materials

The following supporting information can be downloaded at https://www.mdpi.com/article/10.3390/diagnostics14151658/s1, Table S1: Mixed Methods Appraisal Tool (MMAT) used to assess the quality of the studies. Table S2: PRISMA 2020 Checklist. References [5,7,8,9,10,11,12,13,14,15,16,17,18,20,21,22,23,24,26,27,28,29,32] are cited in the Supplementary Materials.
