# Supplementary material for: Systematic Review on the Use of 3D-Printed Models for Planning, Training and Simulation in Vascular Surgery
Source: Diagnostics (Basel). 2024 Jul 31;14(15):1658. doi: 10.3390/diagnostics14151658 (PMC11312310; doi:10.3390/diagnostics14151658)
Supplement: Supplementary file 1 [file diagnostics-14-01658-s001.zip › Supplementary Table S1.pptx]

## Slide 1
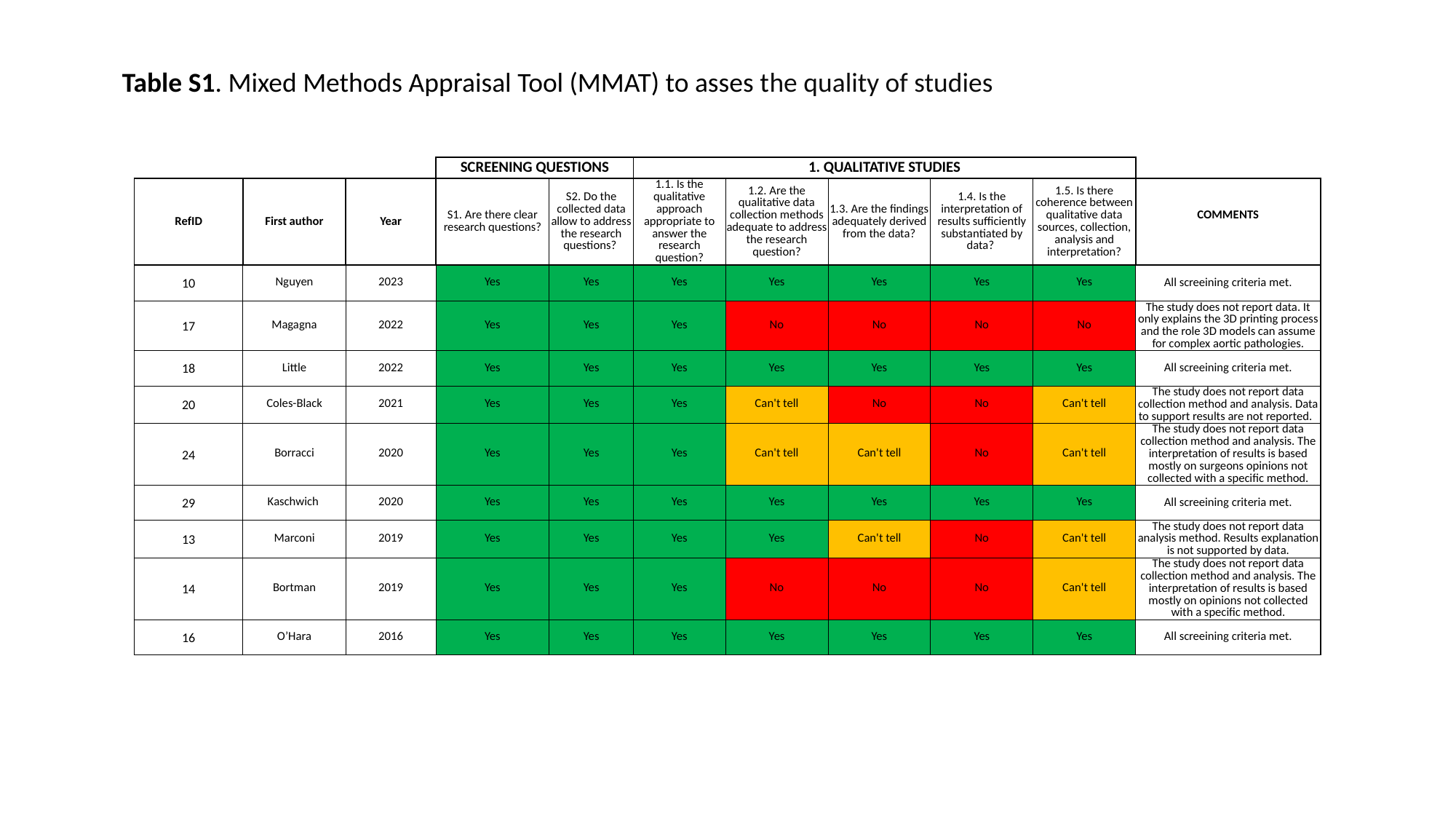

Table S1. Mixed Methods Appraisal Tool (MMAT) to asses the quality of studies
| | | | SCREENING QUESTIONS | | 1. QUALITATIVE STUDIES | | | | | |
| --- | --- | --- | --- | --- | --- | --- | --- | --- | --- | --- |
| RefID | First author | Year | S1. Are there clear research questions? | S2. Do the collected data allow to address the research questions? | 1.1. Is the qualitative approach appropriate to answer the research question? | 1.2. Are the qualitative data collection methods adequate to address the research question? | 1.3. Are the findings adequately derived from the data? | 1.4. Is the interpretation of results sufficiently substantiated by data? | 1.5. Is there coherence between qualitative data sources, collection, analysis and interpretation? | COMMENTS |
| 10 | Nguyen | 2023 | Yes | Yes | Yes | Yes | Yes | Yes | Yes | All screeining criteria met. |
| 17 | Magagna | 2022 | Yes | Yes | Yes | No | No | No | No | The study does not report data. It only explains the 3D printing process and the role 3D models can assume for complex aortic pathologies. |
| 18 | Little | 2022 | Yes | Yes | Yes | Yes | Yes | Yes | Yes | All screeining criteria met. |
| 20 | Coles-Black | 2021 | Yes | Yes | Yes | Can't tell | No | No | Can't tell | The study does not report data collection method and analysis. Data to support results are not reported. |
| 24 | Borracci | 2020 | Yes | Yes | Yes | Can't tell | Can't tell | No | Can't tell | The study does not report data collection method and analysis. The interpretation of results is based mostly on surgeons opinions not collected with a specific method. |
| 29 | Kaschwich | 2020 | Yes | Yes | Yes | Yes | Yes | Yes | Yes | All screeining criteria met. |
| 13 | Marconi | 2019 | Yes | Yes | Yes | Yes | Can't tell | No | Can't tell | The study does not report data analysis method. Results explanation is not supported by data. |
| 14 | Bortman | 2019 | Yes | Yes | Yes | No | No | No | Can't tell | The study does not report data collection method and analysis. The interpretation of results is based mostly on opinions not collected with a specific method. |
| 16 | O’Hara | 2016 | Yes | Yes | Yes | Yes | Yes | Yes | Yes | All screeining criteria met. |

## Slide 2
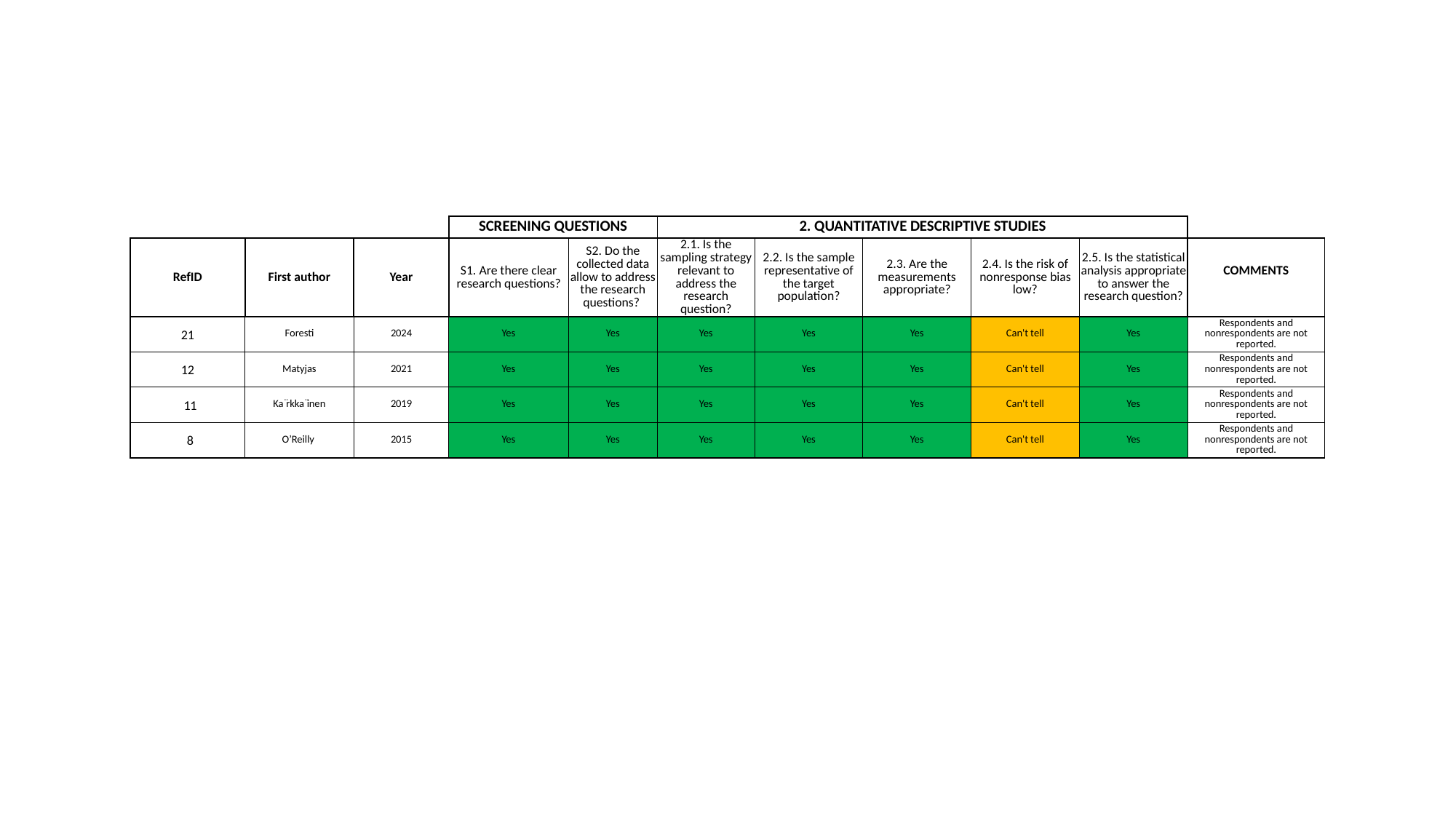

| | | | SCREENING QUESTIONS | | 2. QUANTITATIVE DESCRIPTIVE STUDIES | | | | | |
| --- | --- | --- | --- | --- | --- | --- | --- | --- | --- | --- |
| RefID | First author | Year | S1. Are there clear research questions? | S2. Do the collected data allow to address the research questions? | 2.1. Is the sampling strategy relevant to address the research question? | 2.2. Is the sample representative of the target population? | 2.3. Are the measurements appropriate? | 2.4. Is the risk of nonresponse bias low? | 2.5. Is the statistical analysis appropriate to answer the research question? | COMMENTS |
| 21 | Foresti | 2024 | Yes | Yes | Yes | Yes | Yes | Can't tell | Yes | Respondents and nonrespondents are not reported. |
| 12 | Matyjas | 2021 | Yes | Yes | Yes | Yes | Yes | Can't tell | Yes | Respondents and nonrespondents are not reported. |
| 11 | Ka ̈rkka ̈inen | 2019 | Yes | Yes | Yes | Yes | Yes | Can't tell | Yes | Respondents and nonrespondents are not reported. |
| 8 | O’Reilly | 2015 | Yes | Yes | Yes | Yes | Yes | Can't tell | Yes | Respondents and nonrespondents are not reported. |

## Slide 3
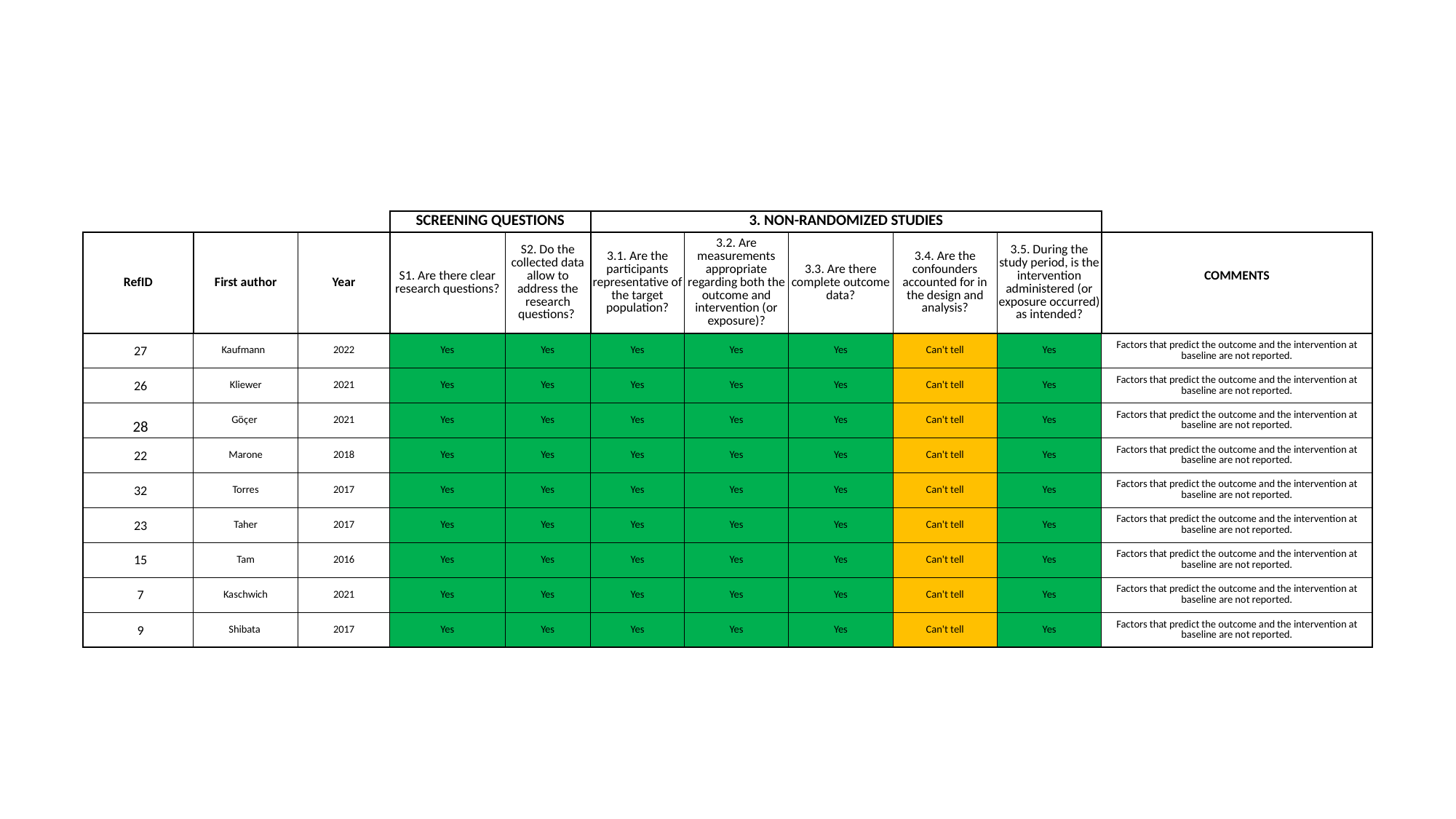

| | | | SCREENING QUESTIONS | | 3. NON-RANDOMIZED STUDIES | | | | | |
| --- | --- | --- | --- | --- | --- | --- | --- | --- | --- | --- |
| RefID | First author | Year | S1. Are there clear research questions? | S2. Do the collected data allow to address the research questions? | 3.1. Are the participants representative of the target population? | 3.2. Are measurements appropriate regarding both the outcome and intervention (or exposure)? | 3.3. Are there complete outcome data? | 3.4. Are the confounders accounted for in the design and analysis? | 3.5. During the study period, is the intervention administered (or exposure occurred) as intended? | COMMENTS |
| 27 | Kaufmann | 2022 | Yes | Yes | Yes | Yes | Yes | Can't tell | Yes | Factors that predict the outcome and the intervention at baseline are not reported. |
| 26 | Kliewer | 2021 | Yes | Yes | Yes | Yes | Yes | Can't tell | Yes | Factors that predict the outcome and the intervention at baseline are not reported. |
| 28 | Göçer | 2021 | Yes | Yes | Yes | Yes | Yes | Can't tell | Yes | Factors that predict the outcome and the intervention at baseline are not reported. |
| 22 | Marone | 2018 | Yes | Yes | Yes | Yes | Yes | Can't tell | Yes | Factors that predict the outcome and the intervention at baseline are not reported. |
| 32 | Torres | 2017 | Yes | Yes | Yes | Yes | Yes | Can't tell | Yes | Factors that predict the outcome and the intervention at baseline are not reported. |
| 23 | Taher | 2017 | Yes | Yes | Yes | Yes | Yes | Can't tell | Yes | Factors that predict the outcome and the intervention at baseline are not reported. |
| 15 | Tam | 2016 | Yes | Yes | Yes | Yes | Yes | Can't tell | Yes | Factors that predict the outcome and the intervention at baseline are not reported. |
| 7 | Kaschwich | 2021 | Yes | Yes | Yes | Yes | Yes | Can't tell | Yes | Factors that predict the outcome and the intervention at baseline are not reported. |
| 9 | Shibata | 2017 | Yes | Yes | Yes | Yes | Yes | Can't tell | Yes | Factors that predict the outcome and the intervention at baseline are not reported. |
